# Multifaceted oncostatin M: novel roles and therapeutic potential of the oncostatin M signaling in rheumatoid arthritis

**DOI:** 10.3389/fimmu.2023.1258765

**Published:** 2023-11-01

**Authors:** Liang Han, Jiahui Yan, Tingting Li, Weiji Lin, Yao Huang, Pan Shen, Xin Ba, Ying Huang, Kai Qin, Yinhong Geng, Huanhuan Wang, Kaifeng Zheng, Yafei Liu, Yu Wang, Zhe Chen, Shenghao Tu

**Affiliations:** ^1^ Department of Integrated Traditional Chinese and Western Medicine, Tongji Hospital, Tongji Medical College, Huazhong University of Science and Technology, Wuhan, China; ^2^ Department of Rheumatology and Immunology, Zhongnan Hospital, Wuhan University, Wuhan, China; ^3^ Department of Nephrology, The First Affiliated Hospital of Zhengzhou University, Zhengzhou, China

**Keywords:** oncostatin M (OSM), rheumatoid arthritis (RA), fibroblast-like synoviocytes (FLS), cytokines, cartilage and bone destruction, CD4^+^ T cell, pannus formation

## Abstract

Rheumatoid arthritis (RA) is a self-immune inflammatory disease characterized by joint damage. A series of cytokines are involved in the development of RA. Oncostatin M (OSM) is a pleiotropic cytokine that primarily activates the Janus kinase (JAK)/signal transducer and activator of transcription (STAT) signaling pathway, the mitogen-activated protein kinase (MAPK) signaling pathway, and other physiological processes such as cell proliferation, inflammatory response, immune response, and hematopoiesis through its receptor complex. In this review, we first describe the characteristics of OSM and its receptor, and the biological functions of OSM signaling. Subsequently, we discuss the possible roles of OSM in the development of RA from clinical and basic research perspectives. Finally, we summarize the progress of clinical studies targeting OSM for the treatment of RA. This review provides researchers with a systematic understanding of the role of OSM signaling in RA, which can guide the development of drugs targeting OSM for the treatment of RA.

## 1 Introduction

Rheumatoid arthritis (RA) is an autoimmune disease of unknown etiology, which is believed to be associated with genetic, environmental, and immunological factors (1). The primary clinical manifestation of RA is symmetrical joint inflammation, which is based on synovitis, bone, and cartilage destruction (2). Cytokines play a crucial role in the pathogenesis of RA and are of paramount importance in RA pathology (3, 4). Cytokines such as tumor necrosis α (TNF-α), interleukin 6 (IL-6), IL-1, IL-17, and IL-10 promote or inhibit the occurrence and development of RA. Fibroblast-like synoviocytes (FLS) and synovial macrophages in the joint cavity of RA patients secrete a large number of pro-inflammatory cytokines and chemokines, which are important mechanisms leading to joint inflammation, bone destruction, and angiogenesis in RA patients (5–7). Therefore, ongoing research is revealing the relationship between cytokines and RA, with the aim of further exploring the molecular mechanisms underlying RA, discovering new disease activity biomarkers, and even finding new targets for treating RA.

As a member of the IL-6 family, oncostatin M (OSM) has gradually received attention for its role and mechanism in the occurrence of rheumatoid arthritis (RA) (8). OSM is a pleiotropic cytokine that participates in the regulation of multiple signaling pathways and plays an important role in the pathogenesis of various autoimmune diseases (9). It has been observed that RA patients have high levels of OSM in their synovial fluid, and further research has found that OSM can promote the occurrence and development of RA through multiple pathways (9, 10). The present paper intends to introduce the research progress of OSM at the current stage from the aspects of OSM and its receptor complex, OSM signal transduction, OSM’s biological functions, and the relationship between OSM and the pathogenesis of RA.

## 2 OSM and OSM receptor complex

The IL-6 cytokine family is one of the largest cytokine families, and the common feature of cytokines in this family is the presence of the signal receptor subunit gp130 in their receptor complexes. These cytokines include IL-6, IL-11, ciliary neurotrophic factor (CNTF), leukemia inhibitory factor (LIF), OSM, IL-12, cardiotrophin-1 (CT-1), cardiotrophin-like cytokine factor 1 (CLCF1), and IL-27 (11). It is worth noting that the receptor complex of the cytokine IL-31 contains a gp130-like receptor (GPL), so some researchers consider IL-31 as part of the IL-6 cytokine family. OSM is an important member of the IL-6 family of cytokines, first discovered by Zarling et al. in the U-937 human lymphoma cell line (12). The human OSM gene is located in the chromosome 22q12.2 region, and the OSM polypeptide transcribed from the human OSM gene includes 252 amino acid residues, with N- and C-terminals consisting of 25 and 32 amino acid residues, respectively. After proteolytic processing, only 195 amino acid residues are retained. The mature human OSM protein has a molecular weight of 28 kDa, and its structure consists of four α-helices arranged in an “up-up-down-down” topology (13–15). OSM is widely expressed *in vivo*, and many immune cells such as T cells, monocytes/macrophages, and neutrophils can express OSM (16–19).

**Figure 1:**
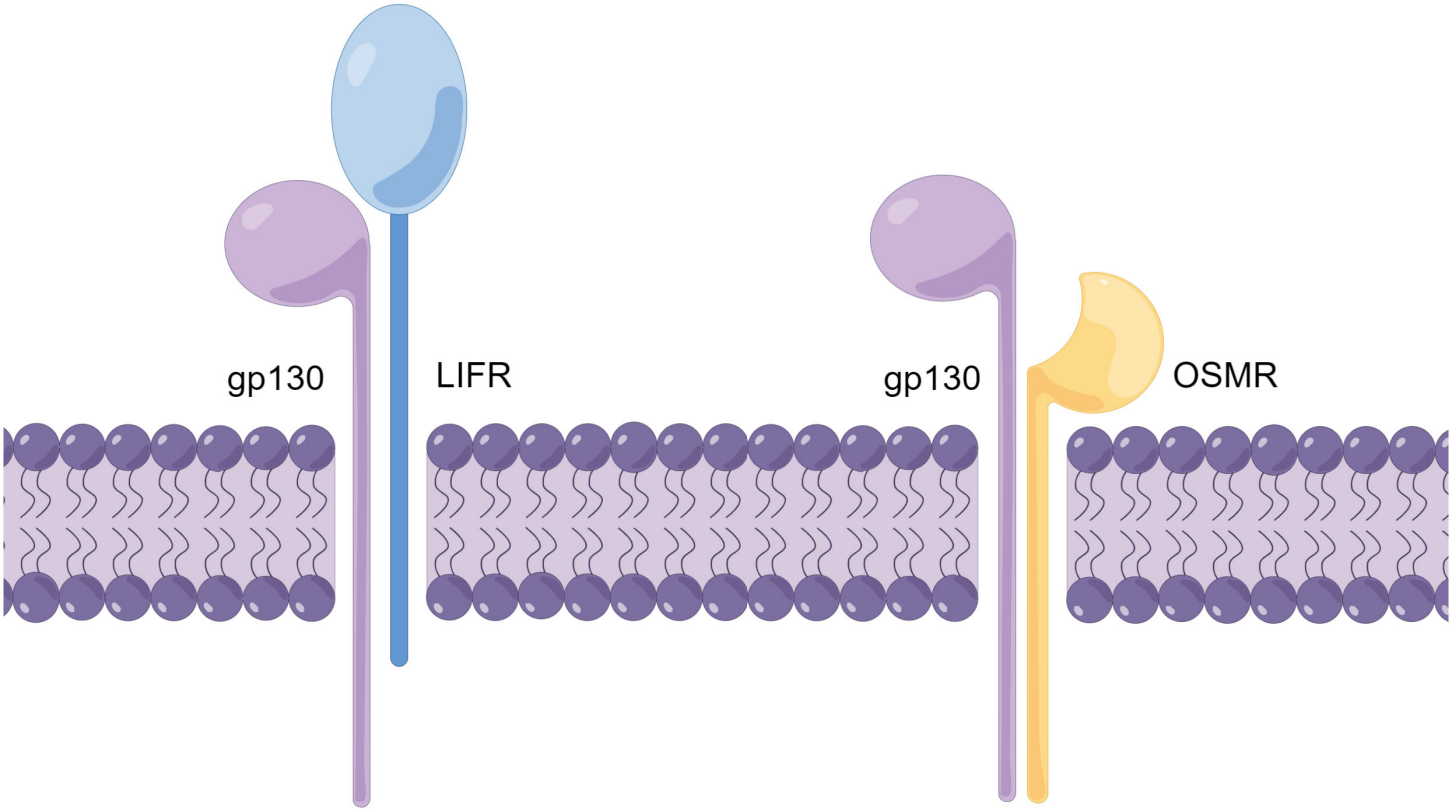
Schematic diagram of the OSM receptor complex structure (By Figdraw.). The OSM receptor complex is a heterodimer and can be classified into two types (Type I and Type II), with different subunits comprising each receptor complex. The Type I OSM receptor complex is composed of gp130 and LIFR, while the Type II OSM receptor complex is composed of gp130 and OSMR. The schematic diagram illustrates the structure of the OSM receptor complex.

As mentioned earlier, the receptor complexes of the IL-6 family of receptors all contain the gp130 subunit (20). The receptor complexes of OSM are all heterodimers, and depending on the different second subunits in the receptor complexes, the receptor complexes can be divided into two types: type I and type II (21). The type I OSM receptor complex is composed of the α subunit gp130 and the β subunit LIFRβ (LIF receptor β subunit), while the type II OSM receptor complex is composed of the a subunit gp130 and the b subunit OSMRb (OSM receptor b subunit) (**Figure 1**) (22). To avoid confusion, in this article, OSMR specifically refers to the β subunit of the type II OSM receptor complex, while OSM receptor refers to both type I and type II OSM receptor complexes.

**Figure 2:**
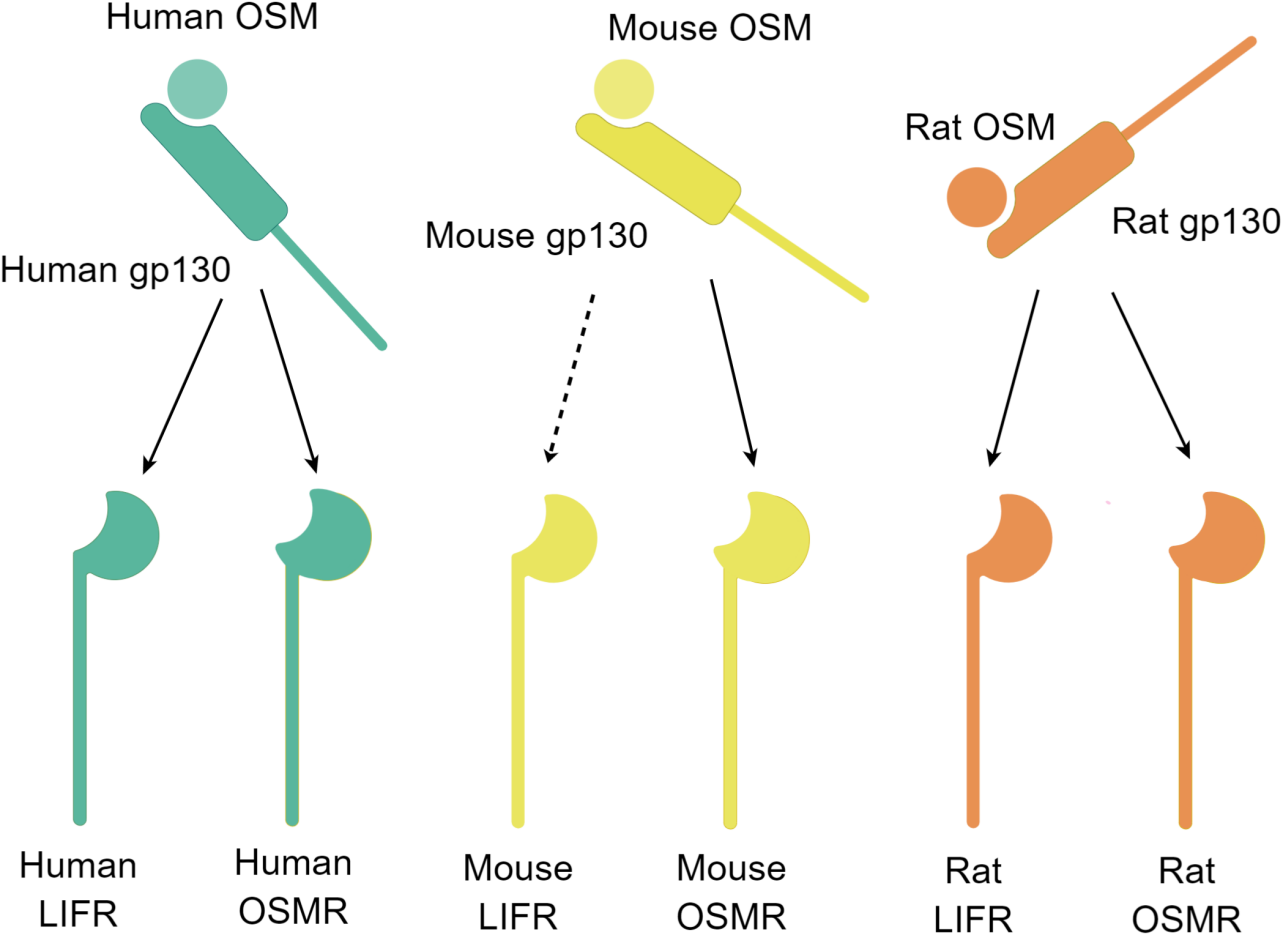
Schematic diagram of the OSM and receptor complex binding (By Figdraw.). Free OSM initially binds to gp130 and subsequently binds to another subunit, LIFR or OSMR. The binding ability of OSM and the two receptors differs among different species. The OSM-gp130 complex in humans and rats can bind to LIFR or OSMR of the same species, whereas in mice, the OSM-gp130 complex can bind to OSMR, and at high concentrations, it can also bind to LIFR (dashed line marked). The schematic diagram depicts the binding of the OSM and receptor complex.

It is worth noting that the two subunits of the OSM receptor complex exist separately in the resting state. When both OSM and the two subunits of the OSM receptor complex are present, OSM first forms a low-affinity heterodimer with gp130, and then this heterodimer recruits OSMR or LIFR and binds to it (9). Further research through computational simulations has identified several key amino acid residues involved in the binding process between OSM and OSMR (23). Additionally, research using computational simulations has revealed that the phenomenon of OSM and LIF sharing the LIFR receptor is related to the structural similarity between OSM and LIF (24). The binding of OSM and the OSM receptor complex is species-specific, and OSM from mice generally only binds to type II OSM receptor complexes. Only high concentrations of mouse-derived OSM can weakly activate LIFR, but there is also a single report that mouse-derived OSM can activate LIFR in mouse osteoblasts (**Figure 2**) (9, 25, 26). In contrast, OSM from rats or humans can bind to type I or type II OSM receptor complexes of the same species (27). Overall, OSMR as a unique subunit in the OSM receptor complex enables type II OSM receptor complexes to activate downstream pathways different from other IL-6 family signaling pathways, thereby exerting its unique biological functions.

## 3 OSM signaling transduction

The two subunits of the OSM receptor, like other members of the IL-6 family of receptors, have transmembrane structures. When the extracellular region binds to OSM, the intracellular region recruits and activates the Janus kinase (JAK) family. After binding to its receptor complex, the OSM receptor complex can activate JAK1 through gp130, and promote the phosphorylation and dimerization of the transcription factor signal transducer and activator of transcription (STAT), which is the downstream of JAK (20). The STAT protein then enters the nucleus and regulates the expression of target genes. In addition, OSM signaling can also trigger the RAS/mitogen-activated protein kinase (MAPK) signaling pathway, the c-Jun N-terminal kinase (JNK)/p38 MAPK signaling pathway, and the phosphatidylinositol 3-kinase/serine-threonine kinase (PI3K/AKT) signaling pathway (28, 29). It is worth noting that the OSM receptor complex of type II OSM receptor complex has the subunit OSMR, which is only present in the OSM receptor, unlike other cytokines in the IL-6 family. Therefore, OSM can exert its unique biological regulatory function through the type II OSM receptor complex. Like type I receptor complexes, type II receptor complexes of OSM can also bind and activate JAK, but the binding affinity of OSM’s type II receptor complex with JAK1 or JAK2 is equivalent, while type I receptor complexes of OSM can bind JAK1 with high affinity (30).

## 4 The physiological functions of OSM signaling

The physiological functions of OSM were first identified in the human lymphoma cell line U-937, and it was subsequently found to have growth-inhibitory effects on melanoma cells, which led to its naming (12). OSM can regulate cell proliferation by controlling cell cycle checkpoints. The cell cycle refers to the entire process between two cell divisions, which can be divided into the mitotic phase and interphase, which is further divided into the G1 phase, S phase, and G2 phase. In eukaryotes, cell cycle checkpoints regulated by cyclins and cyclin-dependent kinases (CDKs) control whether cells can progress from G1 phase to S phase (31). In breast cancer cell lines treated with OSM, the number of cells before S phase significantly increased, while the number of cells in S phase significantly decreased (32). Additionally, OSM treatment altered the levels of cyclins in breast cancer cell lines, indicating that OSM regulates the cell cycle by controlling cyclin levels, causing cells to stay at the cell cycle checkpoint and inhibiting the division of breast cancer cell lines. Furthermore, OSM can inhibit tumor cell division by regulating the expression levels of CDKs in melanoma cell line A375 and liver cancer cell line HepG2, hindering the tumors from progressing through the cell cycle checkpoint (33, 34). However, during liver regeneration, OSM promotes liver cell proliferation by promoting STAT3 phosphorylation (35). These studies suggest that the regulatory effects of OSM on cell proliferation are context-dependent and cannot be generalized.

In addition to its role in regulating cell proliferation, OSM also plays a regulatory role in many physiological processes, including inflammation, immune regulation, and hematopoiesis. As previously mentioned, OSM can activate inflammatory signaling pathways such as JAK/STAT and PI3K/AKT. In human vascular smooth muscle cells and mouse fibroblasts, OSM can also induce the expression of pro-inflammatory cytokine IL-6 (36, 37).

OSM also exhibits complex immunomodulatory effects. OSM has been shown to enhance innate immunity. Treatment of Huh7 liver cancer cells with OSM upregulates the expression of related innate immune molecules, intracellular adhesion molecule-1 (ICAM-1), and IL-15 receptor and enhances interferon-α-induced gene transcription, thereby enhancing interferon-α’s ability to combat hepatitis A and B viruses (38). Additionally, OSM inhibits naive CD4^+^ T cell differentiation into Th17 cells by activating suppressors of cytokine signaling 3 (SOCS3), STAT3, and STAT5 through cytokine signaling transduction (39). These findings demonstrate that OSM regulates both the innate and adaptive immune systems.

OSM is also closely related to hematopoiesis. OSMR^-/-^ mice exhibit reduced numbers of peripheral circulating red blood cells and platelets, as well as a corresponding decrease in megakaryocyte-erythroid progenitor cell (MEP) numbers in the bone marrow (8, 40). During embryonic development in vertebrates, the fetal liver is a natural site for the expansion of hematopoietic stem cells, and in mice, approximately half of the hematopoietic cells reside in the liver during embryonic development. At the end of hematopoiesis, hepatic hematopoietic cells express OSM and act on OSM receptors on the fetal liver stromal cells, promoting the maturation of the fetal liver and the loss of hematopoietic support function (41).

## 5 OSM signaling and diseases

Abnormal levels of OSM are associated with various inflammatory diseases. Compared to healthy controls, OSM levels are elevated in the peripheral blood plasma of patients with coronavirus disease 2019 (COVID-19), and are associated with the severity of the disease (42). Colonic and serum OSM levels are not only elevated in patients with inflammatory bowel diseases (IBD) who experience postoperative recurrence, but also high levels of OSM in colonic tissue often indicate poor prognosis or insensitivity to biologic therapy in IBD patients (43).

Previously, it was mainly believed that OSM mainly affects the development of many diseases by promoting inflammation. For example, researchers have found that lipopolysaccharide (LPS) produced by respiratory microbiota imbalance can promote OSM secretion by macrophages in the respiratory tract, which not only promotes airway inflammation and mucus secretion in patients with severe asthma. However, blocking the OSM signal with OSM-specific antibodies can alleviate asthma-related pathological features without affecting important antibacterial immune responses in the body (44). In addition, OSM can also promote the expression of inflammatory factors CC chemokine ligand (CCL) 2, IL-6, and vascular endothelial growth factor (VEGF) by inducing human aortic adventitial fibroblasts and smooth muscle cells in synergy with LPS, ultimately promoting the development of atherosclerosis (45). However, some studies have shown that OSM can play an anti-inflammatory role in certain diseases. For example, OSM treatment inhibited the expression of TNF-α induced by lipopolysaccharide (LPS) in septic mice and reduced their mortality (46). OSM also exerts an anti-inflammatory effect by promoting macrophage polarization towards the M2 type in adipose tissue (47). Therefore, the regulatory effect of OSM on inflammation is quite complex, and it may have opposite effects in different diseases. It is a pleiotropic cytokine.

In addition, OSM can also affect the progression of diseases by regulating fibrosis through various pathways. Overexpression of OSM in mouse liver tissue leads to upregulation of the expression of the profibrotic cytokine transforming growth factor-β (TGF-β) in liver macrophages, which promotes liver tissue fibrosis (48). Tissue inhibitor of matrix metalloproteinase (TIMP) can inhibit the expression of matrix metallopeptidase (MMP) 1. In cardiac fibroblasts, OSM inhibits the expression of TIMP1, which promotes the deposition of local extracellular matrix, leading to tissue fibrosis (49). Some studies have also found that OSM directly binds to the extracellular matrix, which may increase the stability of the extracellular matrix and promote its deposition, indicating that OSM may directly promote fibrosis (50). The profibrotic effect of OSM may also be beneficial for disease recovery. *In vitro* studies have shown that OSM can accelerate wound healing by promoting collagen and glycosaminoglycan production in fibroblasts at the site of diabetic foot ulcers (51). Further animal experiments have found that local treatment with OSM promotes wound healing in diabetic mice (52).

Recent studies suggest that OSM may have a role in the development and progression of other diseases beyond regulating inflammation and fibrosis. For instance, OSM secreted by T cells and monocytes in the dermis may enhance the sensitivity of sensory neurons to pruritogens, leading to increased itchiness in inflammatory skin lesions (53).

It is evident that OSM, a pleiotropic cytokine, plays a crucial role in the pathogenesis of many inflammatory diseases. Numerous studies have demonstrated the significant involvement of OSM levels in rheumatic diseases. Single nucleotide polymorphisms (SNP) rs22922016 related to OSMR gene has been found to associated to systemic lupus erythematosus (SLE) (54). Moreover, OSM signaling pathway was identified as a potential pathway which related to SLE by analyzing transcriptomic and genome-wide association studies data (55). Systemic sclerosis (SSc), a rheumatic disease characterized by skin thickening, hardening, and visceral fibrosis, is also associated with OSM signaling. Diffuse cutaneous systemic sclerosis (dcSSc) patients showed significantly elevated levels of OSM in their serum, and OSM and OSM-regulated genes were upregulated in dcSSc skin (56). Fibroblasts positive for OSM and pSTAT3 were increased in affected skin compared to unaffected skin. These findings suggest that OSM signaling activation in the skin of dcSSc patients may be one of the causes leading to downstream STAT3 signaling activation (56). In addition to SLE and SSc, there is also a close association between the OSM signaling pathway and RA. Subsequently, this article will focus on the relationship between OSM and the development of RA.

## 6 The clinical correlation of OSM signaling with RA

Hui et al. first investigated the levels of OSM in synovial fluid from RA patients and found that OSM was detected in 18 out of 20 RA samples, while it was not detected in 10 osteoarthritis (OA) samples used as controls. Additionally, they observed a positive correlation between OSM levels and synovial fluid white blood cell counts (57). Similarly, Cawston et al. detected elevated OSM levels only in RA patients but not in OA or healthy control samples (58). Manicourt et al. later reported a positive correlation between OSM levels in synovial fluid from RA patients and the levels of pro-inflammatory cytokines IL-6 and TNF-α, with a larger sample size (59). OSM in the joint cavity of RA patients could be derived from macrophages and neutrophils. Cawston et al. confirmed that CD14^+^ macrophages were the primary source of OSM in synovial tissue from RA patients using immunohistochemistry (58). Cross et al. found that neutrophils from peripheral blood of RA patients rapidly expressed and released OSM upon stimulation with granulocyte-macrophage colony-stimulating factor (GM-CSF), while neutrophils from synovial fluid did not respond to GM-CSF stimulation (18). Considering that OSM is a secreted protein, neutrophils in synovial fluid from RA patients may have already released a significant amount of OSM in the joint cavity. Furthermore, genomic studies have shown that the T allele of the OSMR gene promoter region (rs22922016 locus) has a protective effect on RA patients (54). These findings suggest a close relationship between the OSM signaling pathway and the development of RA.

## 7 Potential effects of OSM signaling on the pathogenesis of RA

**Figure 3:**
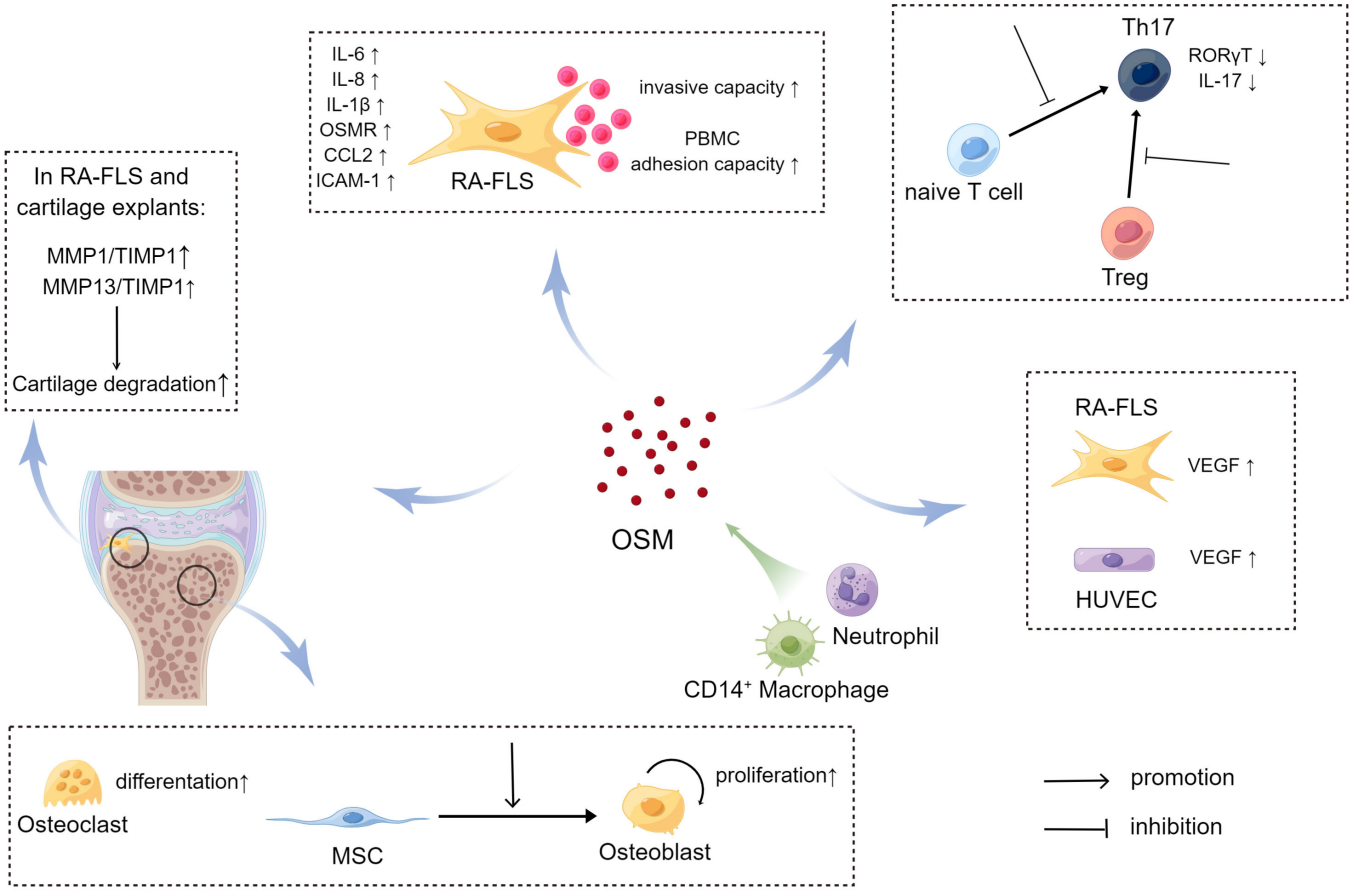
The impact of OSM on signal transduction (upper left) and metabolic profile (lower right) of RA-FLS (By Figdraw.). In RA-FLS, OSM activates JAK adjacent to the OSM receptor upon binding, followed by activation of downstream signaling pathways such as STAT and MAPK signaling, affecting the expression of pathogenic genes such as IL6 and IL8 in the cell nucleus. Tofacitinib inhibits downstream signaling pathways by blocking JAK activation. OSM signaling may activate transcription factor HIF-1α in RA-FLS and promote transcription of downstream glucose transporter (GLUT1) and glycolysis-related genes (HK2, PFKFB3), thereby increasing the ECAR/OCR of cells and promoting a shift in metabolic profile toward glycolysis in RA-FLS.

As previously mentioned, evidence from both tissue and blood samples of RA patients suggests that OSM may be involved in the pathogenesis of RA. In fact, OSM is not just a bystander in the pathogenesis of RA, but it also participates in regulating RA development through various mechanisms (**Figure 3**). The following section will focus on elucidating the molecular biological mechanisms through which OSM may impact RA.

### 7.1 The impact of OSM signaling on RA-FLS

The synovium is a connective tissue that attaches to the surrounding cartilage of the joint and is an essential component of the joint. The synovium not only provides direct physical connections to the musculoskeletal system but also reduces friction between joints by secreting synovial fluid, provides nutrition to other tissues within the joint, and is critical for maintaining normal physiological function (60). Histologically, the human synovium can be divided into two layers: the synovial lining layer near the joint cavity and the sublining layer beneath the lining layer. Under normal conditions, the synovial lining layer consists of only 2-3 cell layers, mainly composed of synovial macrophages (SMs) and FLSs. Synovitis is one of the major pathological changes in RA, and FLSs play a key role in the pathogenesis of RA synovitis (61). After being stimulated by a series of pro-inflammatory cytokines, such as TNF-α, in the joints of RA patients, RA-FLS continue to produce inflammatory factors to maintain chronic inflammation in the synovium, while also proliferating, migrating, and invading to destroy joint cartilage tissue (62, 63).

**Figure 4:**
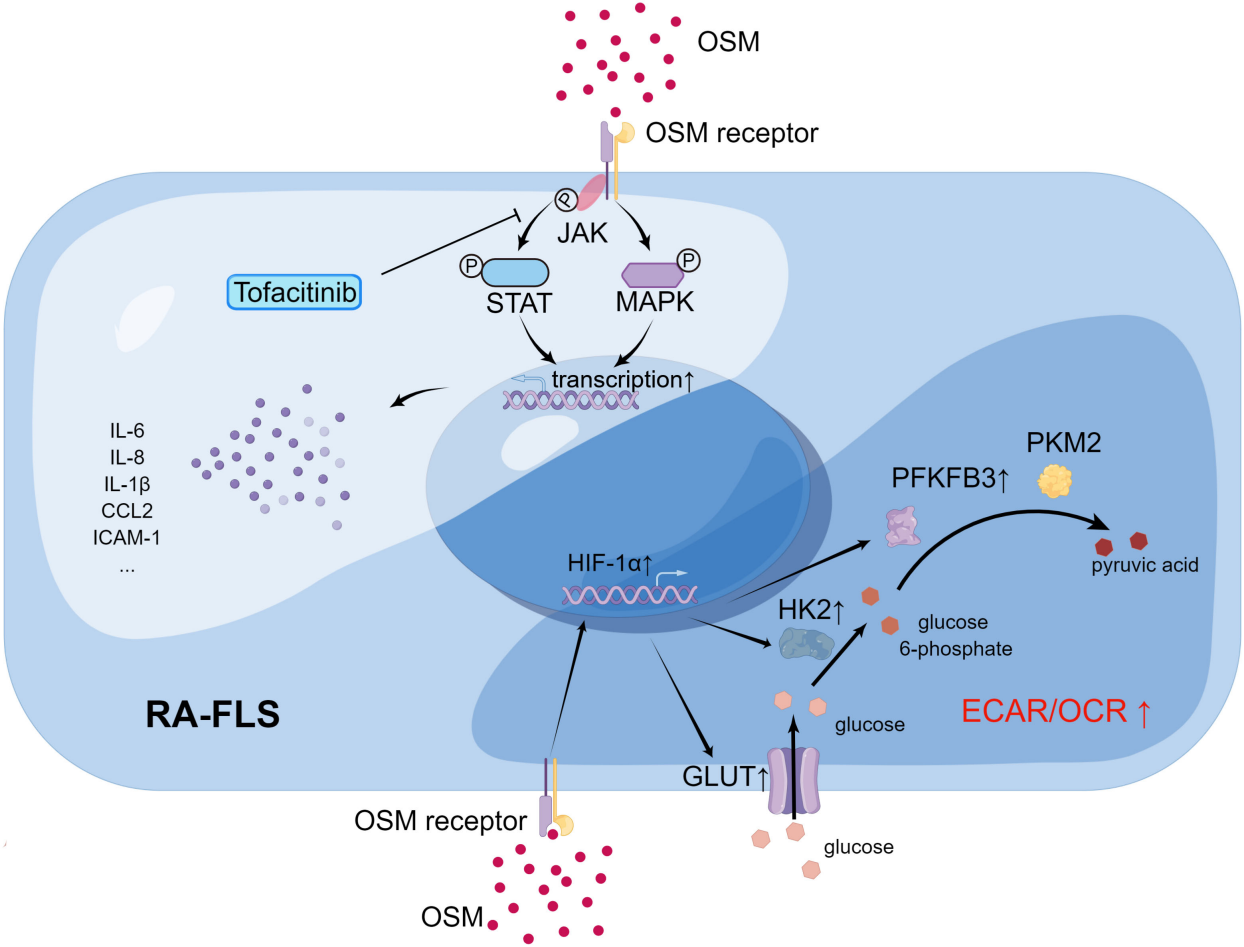
The source and pathogenic role of OSM in rheumatoid arthritis (RA) joints (By Figdraw.). In RA joints, CD14^+^ macrophages and neutrophils are potential major sources of Oncostatin M (OSM), which plays a regulatory role in RA-FLS, bone and cartilage, pannus formation, and T cell differentiation. Through these pathways, OSM may impact the development of RA. In the case of RA-FLS, OSM promotes secretion of relevant cytokines, increases the invasive ability of RA-FLS, and enhances adhesion of RA-FLS and PBMC to promote joint damage. OSM also affects bone and cartilage metabolism. For instance, OSM enhances differentiation of MSC to osteoblasts, promotes the proliferation of osteoblasts, and stimulates the differentiation of osteoclasts. Additionally, OSM increases the secretion of MMPs, which further enhances cartilage degradation. OSM inhibits the differentiation of naive T cells to Th17 cells by suppressing the expression of RORγT. It also reduces the conversion of Treg to Th17 and decreases the secretion of IL17 from Th17 cells. Moreover, OSM promotes the secretion of VEGF, which facilitates formation of pannus.

Studies have shown that OSM can affect RA-FLS through multiple pathways. Migita et al. found *in vitro* that OSM treatment promoted JAK/STAT pathway activation by inducing JAK and STAT phosphorylation in RA-FLS, and also induced RA-FLS to secrete the pro-inflammatory cytokine IL-6 by activating the MAPK pathway (64). The JAK inhibitor tofacitinib was able to inhibit the aforementioned changes induced by OSM. A more in-depth study by Hanlon et al. found that OSM treatment not only promoted RA-FLS to secrete IL-6, but also promoted RA-FLS to secrete the pro-inflammatory cytokines IL-8, chemokine C-C Motif Chemokine Ligand 2 (CCL2), and adhesion molecule ICAM-1 (65, 66). Using mouse FLS, Goff et al. demonstrated that OSM can synergistically enhance the expression of IL-6 with other pro-inflammatory cytokines, such as TNF-α and IL-1β (67). Furthermore, OSM treatment increased the expression of OSMR and IL-1β receptors (IL-1βR) in human RA-FLS and wild-type mouse FLS, which further activated the inflammatory signaling pathway in FLS through positive feedback. Therefore, OSM stimulation of RA-FLS by inducing the secretion of a series of pro-inflammatory cytokines and chemokines aggravates synovitis and angiogenesis in RA (**Figure 4**). In addition to inducing RA-FLS to secrete cytokines and their receptors, OSM also increases the invasive ability of RA-FLS and the adhesion of peripheral blood mononuclear cells (PBMCs) to RA-FLS (65).

Interestingly, OSM also causes changes in the metabolic pathways of RA-FLS, known as metabolic reprogramming (**Figure 4**). Oxygen consumption rate (OCR) and extracellular acidification rate (ECAR) are metrics used to measure mitochondrial respiration and glycolysis, respectively. After OSM treatment, the ECAR/OCR ratio in RA-FLS cells increased, and OSM induced the expression of glucose transporters (GLUT)-1, hexokinase 2 (HK2), 6-phosphofructo-2-kinase/fructose-2,6-biphosphatase 3 (PFKFB3), and hypoxia-inducible factor (HIF)-1α in RA-FLS, indicating that OSM promotes glycolytic metabolism in RA-FLS (65). It is worth noting that most tumor cells also rely on glycolysis to produce energy, and glycolysis-related metabolic intermediates promote the invasion and metastasis of tumor cells (68). As mentioned earlier, OSM treatment also enhances the invasive ability of RA-FLS, which is similar to tumor cells. Therefore, OSM is likely to enhance the intra-articular invasive ability of RA-FLS by promoting glycolytic metabolism in RA-FLS.

### 7.2 OSM signaling and bone homeostasis and cartilage metabolism

Under normal circumstances, a balance between osteoblast-mediated bone formation and osteoclast-mediated bone resorption maintains bone homeostasis. However, in patients with rheumatoid arthritis (RA), increased osteoclast generation and activity, as well as suppressed osteoblast-mediated bone formation, disrupts the normal bone homeostasis, leading to bone and cartilage destruction and bone loss (69). OSM has biological functions that regulate bone metabolism, which can be seen in its regulation of mesenchymal stem cell osteogenic differentiation and its modulation of osteoblast and osteoclast-mediated bone formation and resorption.

Studies have found that OSM is capable of promoting mesenchymal stem cell differentiation into osteoblasts. It has been shown that treatment with OSM can activate JAK, MAPK and other signaling molecules, promoting osteogenic differentiation of human or mouse adipose-derived mesenchymal stem cells (70, 71). Similarly, in mouse fibroblast cell line C3H10T1/2, OSM treatment can induce osteogenic differentiation and promote terminal calcium deposition and mineralized nodule formation (72). Synovial-derived mesenchymal stem cells (SMSCs) in synovial fluid also have the potential to differentiate into osteoblasts, and studies have attempted to induce SMSCs to differentiate in a directed manner for the treatment of joint bone defects (73). Therefore, it is reasonable to assume that higher levels of OSM in RA joints can promote osteogenic differentiation of SMSCs.

OSM also has the ability to promote the proliferation and osteogenic differentiation of osteoblasts. OSM can promote osteogenic differentiation of mouse embryo osteoblast precursor cells MC3T3-E1 by regulating CCL2 (74). Additionally, OSM can promote osteoclast differentiation. Studies have found that OSM, in combination with pro-inflammatory cytokines TNF-α or IL-1β, can activate the RANKL-RANK signaling pathway and significantly increase the number of TRAP-positive cells (i.e. osteoclasts) in mouse joints (75).

The aforementioned studies collectively indicate that OSM has a promoting effect on both bone formation and bone resorption. Therefore, *in vivo* studies exploring the comprehensive effects of OSM on bone formation and resorption are necessary. Previous studies have found that injection of OSM into mice with tibial injury can induce intra-membrane bone formation, suggesting that OSM promotes bone formation in mice (76). However, in a mouse model of arthritis, adenoviral overexpression of joint OSM promotes bone erosion, suggesting that excessive OSM can also contribute to pathological bone destruction (8, 75). Taken together, these two studies suggest that OSM may overall promote bone destruction in the inflammatory environment of arthritis.

Joint damage in RA patients is also closely related to cartilage destruction. High levels of MMPs in the joint cavity of RA patients can degrade the extracellular matrix and absorb cartilage, thereby promoting joint destruction (77). OSM can also regulate cartilage metabolism. Collagen fibers and proteoglycans are the main structural substances of joint cartilage. Clinical studies have found a positive correlation between OSM levels in the synovial fluid of RA patients and the levels of antigenic keratan sulfate (Ag KS) and D-pyridinoline (D-Pyr), suggesting that OSM may promote the degradation of proteoglycans and collagen in joint cartilage of RA patients (59). *In vitro* experiments have shown that OSM treatment of pig joint cartilage explants increases the release of proteoglycans while inhibiting synthesis (78). MMPs can degrade cartilage by cleaving collagen. Using co-cultures of RA-FLS and normal human joint cartilage explants, Fearon et al. found that OSM treatment increased the levels of MMP1 and the MMP inhibitor TIMP1, both of which can degrade the extracellular matrix (79). However, the ratio of MMP1 to TIMP1 increased after OSM treatment, and compared to the use of a single cytokine, the combined use of OSM and IL-1β significantly increased the ratio of MMP1 to TIMP1 and MMP13 to TIMP1. These results suggest that in an inflammatory environment, OSM can promote cartilage degradation.

### 7.3 OSM signaling and pannus formation

Pannus formation is another major pathological change in RA. As synovitis progresses in RA patients, a large number of immune cells infiltrate, the synovial lining layer proliferates and transforms into pannus, extending and adhering to cartilage, promoting the degradation of cartilage and bone tissue (80). Pannus consists of neovascularization, proliferating synovial cells, immune cells, and fibrinoid deposits, and angiogenesis mediated by cell factors such as VEGF is one of the important factors in angiogenesis formation (81, 82). Hanlon et al. found that OSM treatment not only promotes RA-FLS and human umbilical vein endothelial cells to secrete pro-inflammatory cytokines such as IL-6 and CCL2, but also promotes the expression of VEGF (65). Furthermore, OSM and IL-1β combined treatment significantly upregulated the levels of VEGF in the co-culture supernatant of RA-FLS and normal human joint cartilage explants (79). Therefore, it can be seen that OSM can enhance angiogenesis by promoting the expression of VEGF, thus exacerbating the formation of angiogenesis in synovial tissue of RA.

### 7.4 OSM signaling and CD4^+^ T cells

The imbalance of CD4^+^ T cell subsets also plays a significant role in the pathogenesis of RA. Among the various CD4^+^ T cell subsets, the imbalance between the pro-inflammatory Th17 subset and the immunosuppressive regulatory T (Treg) subset is closely related to the development and progression of RA. Currently, there are not many studies on the effects of OSM on CD4^+^ T cell differentiation and function. One study found that OSM treatment inhibited the differentiation of CD4^+^ T cells from mouse spleen into Th17 cells, suppressed Th17-related cytokine IL-17, and the Th17-specific transcription factor retinoic acid-related orphan receptor γt (RORγt) expression (39). Further research found that OSM inhibited CD4^+^ T cell differentiation into Th17 cells by activating STAT3, STAT5, and suppressor of cytokine signaling (SOCS) 3 signaling in a dose-dependent manner. In addition, OSM also inhibited the proliferation of Th17 cells and the conversion of Treg cells into Th17 cells. Similarly, Shirshev et al. found that combined treatment with sex hormones and OSM increased the ratio of Treg cells in CD4^+^ T cells derived from PBMCs, which helps to maintain pregnancy-related immune tolerance (83).

From the perspective of the effect of OSM on CD4^+^ T cell subset differentiation, OSM can alleviate RA inflammatory responses. However, this contradicts many clinical studies and animal experiments. Considering that OSM is a pleiotropic cytokine, we speculate that although OSM inhibits Th17 cell differentiation, it also has the effect of promoting the secretion of inflammatory cytokines, promoting bone destruction and angiogenesis in RA. Overall, OSM still promotes the pathogenesis of RA.

## 8 The OSM signaling pathway in arthritis animal models

The aforementioned studies suggest that OSM is a pleiotropic cytokine, thus it is necessary to examine its overall regulatory effect in animal models of arthritis. Currently, most studies on animal models of arthritis suggest that OSM promotes the onset and progression of arthritis. In mice with collagen-induced arthritis (CIA), the mRNA levels of OSM in joint tissues are elevated, and the severity of arthritis and histopathological changes can be improved by neutralizing OSM with antibodies (84). In mice with pristane-induced arthritis (PIA), preemptive OSM neutralization with antibodies can prevent the onset of arthritis symptoms (84). Local overexpression of OSM in mouse joints using adenoviral vectors leads to synovial hyperplasia, mononuclear cell infiltration, and cartilage damage (85). In mice with antigen-induced arthritis (AIA), the expression levels of OSM and its type I and II receptors are elevated (67). In a rat model of peptidoglycan-polysaccharide (PGPS)-induced arthritis, OSM levels in the synovial membrane are higher than those in the control group, and the levels of OSM in the knee joints of rats are positively correlated with the Krenn score (used to evaluate synovitis histopathology) and the Mankin score (used to evaluate cartilage damage) (86). However, some studies have also shown that OSM can improve joint lesions in animal models of arthritis. Wahl et al. used four different monoclonal antibodies against collagen and LPS to construct a mouse model of arthritis and found that the mice in the arthritis group exhibited joint pathology changes such as angiogenesis, cartilage and bone erosion, while the joint pathology changes in the OSM-treated group were minimal (46). This result is obviously contradictory to other relevant studies. However, it is worth noting that Wahl et al. used human OSM in their study, which cannot bind to mouse OSMR and activate downstream signaling pathways, but can bind to receptors such as LIFR in mice and exert anti-inflammatory effects (9, 29).

## 9 Research on targeted OSM signaling therapy for patients with RA

There have been studies attempting to treat RA patients by antagonizing OSM signaling. Choy et al. attempted to construct a humanized monoclonal antibody against OSM (GSK315234) and conducted a Phase II clinical trial to test its efficacy for treating RA patients (87). The results showed that RA patients had good tolerance to GSK315234, and common adverse events included aggravation of RA, elevated alanine aminotransferase, fever, headache, hypertension, and diarrhea, but no one withdrew from the study due to adverse events. However, the therapeutic effect of GSK315234 on RA was not very satisfactory. In the multi-dose, intravenous infusion trial, GSK315234 improved the disease activity score using 28 joint counts (DAS28) in RA; in the single-dose, intravenous infusion control trial, there was no difference in DAS28 between the treatment group and the placebo group; in the single-dose, subcutaneous injection trial, there was a statistically significant difference in DAS28 between the treatment group and the placebo group. In all three clinical trials, there was no statistically significant difference in erythrocyte sedimentation rate or C-reactive protein between the treatment group and the placebo group. Pharmacokinetic and pharmacodynamic analyses revealed that the low affinity of GSK315234 for OSM may be the main reason for its unsatisfactory therapeutic effect on RA.

Besides GSK315234, there are other monoclonal antibodies targeting the OSM signaling pathway. GSK2330811, another anti-OSM monoclonal antibody, has demonstrated sufficient affinity and good tolerability in healthy individuals and has been explored for the treatment of SSc and Crohn’s disease (88). In a completed multicenter, randomized, double-blind, placebo-controlled clinical trial, GSK2330811 was administered at doses of 100 mg or 300 mg weekly for 12 weeks in the treatment of SSc. However, the trial found that changes in inflammation or fibrosis biomarkers in SSc subjects following GSK2330811 treatment were not ideal. Additionally, GSK2330811 led to dose-dependent decreases in hemoglobin or platelet counts in 39% and 26% of the subjects, respectively. Overall, GSK2330811 does not appear to be an ideal choice for treating SSc. Another multicenter, randomized clinical trial (NCT04151225), which aimed to use GSK2330811 for the treatment of Crohn’s disease, has been canceled by the sponsor due to a potentially narrow therapeutic window, and no relevant study data have been disclosed.

There are also studies attempting to target OSMR (OSM receptor β subunit) for the development of monoclonal antibodies to treat related diseases. Vixarelimab, also known as KPL-716, is a monoclonal antibody developed against OSMR. It has shown significant reduction in skin itchiness and improvement in quality of life caused by prurigo nodularis in clinical trials (89). Vixarelimab also demonstrated notable improvements in excessive skin keratinization and nodules in prurigo nodularis subjects. Furthermore, compared to the placebo group, the vixarelimab-treated group did not exhibit an increased incidence of overall infections, immune reactions, liver function abnormalities, hematological changes, malignancies, injection site reactions, or drug-related adverse events related to cardiac toxicity. However, the vixarelimab-treated group had a higher incidence of upper respiratory tract infections. Overall, vixarelimab, targeting OSMR, has shown significant efficacy and relatively sufficient drug safety for the treatment of prurigo nodularis, making it a potential emerging therapy for this condition. The relevant information of existing monoclonal antibodies targeting OSM has been summarized in **Table 1**.

**Table 1:** Monoclonal antibody drugs targeting Oncostatin M signaling.

Name	Target	Diseases	National Clinical Trial number
GSK315234	OSM	rheumatoid arthritis	NCT00674635
GSK2330811		systemic sclerosis	NCT03041025
		Crohn's disease	NCT04151225
Vixarelimab/ KPL-716	OSMR	prurigo nodularis	NCT03816891

OSM, Oncostatin M; OSMR, Oncostatin M rreceptor β subunit.

It is worth mentioning that researchers are also developing small molecule drugs targeting OSM and OSMR. SMI-10B is a small molecule drug predicted through screening to target the OSM signaling pathway (90). The drug developers claim that further experiments have shown that this small molecule can significantly reduce OSM-induced STAT3 phosphorylation in cancer cells. Recently, additional unbiased molecular dynamics studies have revealed that SMI-10B can spontaneously bind to OSM (91). Compared to monoclonal antibodies, small molecule inhibitors, as clinical drugs, generally exhibit lower toxicity and antigenicity, as well as better patient compliance when administered orally. Therefore, it is promising to expect the use of small molecule inhibitors targeting the OSM signaling pathway in RA or other related diseases, although further clinical trials are needed to evaluate their therapeutic efficacy.

## 10 Conclusions

OSM, a secreted cytokine, can regulate JAK/STAT, RAS/MAPK, JNK/p38 MAPK, and PI3K/AKT signaling pathways. OSM can regulate physiological processes such as cell proliferation, inflammation, immune response, and hematopoiesis, and is associated with the pathogenesis of various diseases. In RA patients, the gene polymorphism of OSMR is related to disease activity, and OSM levels in synovial fluid and synovium are higher than those in healthy controls. Existing research indicates that OSM mainly affects the occurrence and development of RA by promoting the secretion of pro-inflammatory cytokines and chemokines by RA-FLS, enhancing the invasive ability of RA-FLS, promoting the adhesion of other immune cells to RA-FLS, regulating bone formation and bone destruction in RA joints, promoting angiogenesis, and regulating the differentiation of CD4^+^ T cells. Although OSM has pleiotropic effects, considering clinical studies and animal models of arthritis, it is clear that OSM overall promotes the occurrence and development of RA.

However, it should be noted that the binding of OSM to its receptor has species specificity, which has affected some previous related studies. Currently, research on RA still cannot avoid animal models of arthritis, and the issue of OSM species specificity will be difficult to avoid in the future. Therefore, future researchers should provide detailed information on the species of the reagents used in experiments, and the interpretation of research results should also consider the species specificity of OSM.

Although the clinical study of the anti-OSM monoclonal antibody GSK315234 for treating RA patients did not achieve good results, this is related to the low affinity of GSK315234 for OSM. Future clinical trial research should learn from this lesson and develop anti-OSM antibodies and small molecule inhibitors with high affinity for use in clinical trials to better explore the potential of antagonizing OSM signaling for treating RA and provide new tools for clinical drug treatment of RA.

## Author contributions

LH: Data curation, Investigation, Writing – original draft. JY: Investigation, Visualization, Writing – review & editing. TL: Investigation, Visualization, Writing – review & editing. WL: Investigation, Writing – review & editing. YaH: Writing – review & editing, Funding acquisition. PS: Writing – review & editing, Funding acquisition. XB: Writing – review & editing. YiH: Writing – review & editing, Funding acquisition. KQ: Writing – review & editing, Funding acquisition. YG: Visualization, Writing – review & editing. HW: Visualization, Writing – review & editing. KZ: Visualization, Writing – review & editing. YL: Writing – review & editing. YW: Writing – review & editing. ZC: Writing – review & editing, Funding acquisition. ST: Supervision, Writing – review & editing, Funding acquisition.
